# Anatomic survey of seeding in Alzheimer’s disease brains reveals unexpected patterns

**DOI:** 10.1186/s40478-021-01255-x

**Published:** 2021-10-11

**Authors:** Barbara E. Stopschinski, Kelly Del Tredici, Sandi-Jo Estill-Terpack, Estifanos Ghebremedhin, Fang F. Yu, Heiko Braak, Marc I. Diamond

**Affiliations:** 1 Center for Alzheimer’s and Neurodegenerative Diseases, Peter O’Donnell Jr. Brain Institute, NL10.120, University of Texas Southwestern Medical Center, 6000 Harry Hines Blvd., Dallas, TX 75390 USA; 2 Clinical Neuroanatomy Section/Department of Neurology, Center for Biomedical Research, University of Ulm, Ulm, Germany; 3 Institute of Clinical Neuroanatomy, J. W. Goethe University, Frankfurt am Main, Germany; 4 Department for Radiology, Neuroradiology Division, University of Texas Southwestern Medical Center, Dallas, TX USA

**Keywords:** Alzheimer’s disease, AT8, FRET biosensor, Neurofibrillary tangles, Prion propagation, Tau seeding, NFT staging

## Abstract

Tauopathies are heterogeneous neurodegenerative diseases defined by progressive brain accumulation of tau aggregates. The most common tauopathy, sporadic Alzheimer’s disease (AD), involves progressive tau deposition that can be divided into specific stages of neurofibrillary tangle pathology. This classification is consistent with experimental data which suggests that network-based propagation is mediated by cell–cell transfer of tau “seeds”, or assemblies, that serve as templates for their own replication. Until now, seeding assays of AD brain have largely been limited to areas previously defined by NFT pathology. We now expand this work to additional regions. We selected 20 individuals with AD pathology of NFT stages I, III, and V. We stained and classified 25 brain regions in each using the anti-phospho-tau monoclonal antibody AT8. We measured tau seeding in each of the 500 samples using a cell-based tau “biosensor” assay in which induction of intracellular tau aggregation is mediated by exogenous tau assemblies. We observed a progressive increase in tau seeding according to NFT stage. Seeding frequently preceded NFT pathology, e.g., in the basolateral subnucleus of the amygdala and the substantia nigra, pars compacta. We observed seeding in brain regions not previously known to develop tau pathology, e.g., the globus pallidus and internal capsule, where AT8 staining revealed mainly axonal accumulation of tau. AT8 staining in brain regions identified because of tau seeding also revealed pathology in a previously undescribed cell type: Bergmann glia of the cerebellar cortex. We also detected tau seeding in brain regions not previously examined, e.g., the intermediate reticular zone, dorsal raphe nucleus, amygdala, basal nucleus of Meynert, and olfactory bulb. In conclusion, tau histopathology and seeding are complementary analytical tools. Tau seeding assays reveal pathology in the absence of AT8 signal in some instances, and previously unrecognized sites of tau deposition. The variation in sites of seeding between individuals could underlie differences in the clinical presentation and course of AD.

**Supplementary Information:**

The online version contains supplementary material available at 10.1186/s40478-021-01255-x.

## Introduction

Tauopathies are a heterogeneous group of neurodegenerative diseases defined by progressive brain accumulation of tau aggregates [35]. Sporadic Alzheimer’s disease (AD) is the most common, and is uniquely defined by coexistent tau and amyloid β pathology. AD neuropathology includes intraneuronal somatic and axonal pretangles and neurofibrillary tangles (NFTs), neuropil threads (NTs), extraneuronal ghost tangles, and amyloid β plaques. Tau pathology progresses in a defined and characteristic pattern, allowing AD classification into different stages that correlate with antemortem clinical presentation [4].

Aggregated tau protein is often phosphorylated [35], and the anti-phospho-tau monoclonal antibody AT8 [26] is typically used for detection and staging. AT8 binds phospho-serine 202 and phospho-threonine 205 on aggregated tau protein, and marks AD intraneuronal pathology (pretangles and NFTs) [38]. AT8 signal increases with disease progression and allows the definition of NFT stages [3, 6]. In NFT stage I, AT8 marks selected brainstem nuclei and the transentorhinal cortex (TRE). In NFT stage II, AT8 marks the entorhinal cortex (EC) in the parahippocampal gyrus. In NFT stage III, AT8 marks the CA1 region of the hippocampus, and neocortical regions of the temporal neocortex adjacent to the TRE. In NFT stages IV and V, AT8 marks neocortical regions including the superior temporal gyrus (STG), and in NFT stage VI it marks primary neocortical areas such as the visual cortex (VC). At NFT stages III to IV, more than 50% of individuals have signs of mild cognitive impairment, whereas at NFT stages V and VI more than 90% of individuals exhibit signs of moderate to severe dementia [26]. The severity of AD dementia correlates with the extent of postmortem tau pathology [41, 56]. Additionally, longitudinal tau PET imaging has confirmed the progression of tau pathology along NFT stages, and its correlation with neuronal dysfunction and neurodegeneration [24, 27, 36, 44].

Progressive tau aggregation in AD occurs in patterns consistent with neural networks [4]. Recent data from in vitro and in vivo experimental systems is consistent with trans-neuronal spread of pathology similar to prion disease, in which pathological species move from cell to cell, serving as templates to convert native tau into a pathogenic aggregation-prone form, and thereby propagating tau pathology among connected brain regions [10, 11, 47, 48]. It is unknown whether this mechanism underlies progression in humans, however the presence of soluble, non-aggregated pretangle pathogenic tau “seeds” in human brain that anticipate the development of NFT pathology is very consistent with this idea [20, 28].

To detect tau seeding in biological samples, we previously developed a sensitive and specific cell-based “biosensor” seeding assay, in which the tau repeat domain containing a single disease-associated mutation (P301S) is fused to complementary fluorescent proteins (e.g., cyan/yellow; cerulean/clover; clover/ruby), and expressed in cells of choice. The fusion proteins aggregate upon exposure to tau seeds, which is quantified by flow cytometry [19, 25]. In a transgenic mouse model, the seeding assay scored positive many months before histopathology or insoluble tau protein could be observed [25]. Further, in fresh frozen brain tissue from individuals with AD, the assay also detected seeding prior to neuropathological changes [14, 25]. We subsequently refined the method to quantify seeding in fixed brain sections, which was equally reliable [30]. In fixed tissues from multiple AD patients at different NFT stages, we observed tau seeding first in the TRE and EC rather than in the locus coeruleus (LC), as we had previously hypothesized based on AT8 histopathology [28]. All prior analyses have been confined to regions known to contain NFT pathology in AD. The extent of tau seeding across widespread brain regions, however, is unknown. In this work we have used an optimized biosensor cell line (TauRD(P301S)v2H) [23] and we have tested for seeding across a large cross-section of brain regions with and without known NFT pathology. We have generated a map of AD brain across NFT stages. This has revealed surprising patterns, and a new type of cellular tau pathology.

## Methods

### Generation of biosensor cell line (TauRD(P301S)v2H)

A second generation of high sensitivity biosensor cells termed v2H has recently been produced [23]. Using the previously described lentiviral FM5-YFP plasmid [47], we inserted the tau segment 246 to 378 with the P301S mutation, replaced the human ubiquitin C (Ubc) promoter with a human cytomegalovirus (CMV) promoter, and replaced the YFP sequence with an mCerulean3 or mClover3 coding sequence. To reduce translation read through of the tau ATG start site and increase transgene expression, the sequence upstream of tau was modified to encode an optimal Kozak sequence (5’-GCCACCACCATGGCC-3’). The GCC after the ATG start codon encodes the amino acid A246 in tau. The sequence linking the tau segment and the coding sequence of the fluorophore (mCerulean3 or mClover3) was optimized to the following sequence: 5’- GSAGSAAGSGEF-3’.

To create the v2H line, low passage HEK293T cells (P5) were thawed and passaged with antibiotic free media twice before co-administration of P301S 246–378 tau-mCerulean3 tau-mClover3 lentivirus. After four passages, single cells were isolated via fluorescence activated cell sorting (FACS) based on low, intermediate, and high brightness levels for both mCerulean3 and mClover3. Monoclonal colonies were cultured to high cell number and tested by seeding assays with recombinant fibrils and AD lysate. The v2H line was chosen for low background signal and high sensitivity, and used in subsequent seeding experiments as a next-generation biosensor based on previously established protocols [25].

### Culture of biosensor cells

Stable monoclonal v2H FRET biosensor cells were grown in complete media: Dulbecco’s Modified Eagle’s Medium (DMEM) (Gibco) with 10% fetal bovine serum (Sigma), 1% penicillin/streptomycin (Gibco) and 1% Glutamax (Gibco). Cells were cultured and passaged at 37 °C, 5% CO^2^, in a humidified incubator. Dulbecco’s phosphate buffered saline (Life Technologies) was used for washing the cells prior to harvesting with 0.05% Trypsin–EDTA (Life Technologies).

### Mouse breeding for positive and negative controls

All experiments involving animals were approved by the University of Texas Southwestern Medical Center Institutional Animal Care and Use Committee (IACUC). All mice were housed under a 12 h light/dark cycle, and were provided food and water ad libitum. We used tau KO mice containing a GFP-encoding cDNA integrated into exon 1 of the MAPT gene as a negative control. These mice were obtained from Jackson Laboratory and maintained on a C57BL/6 J background.

As a positive control, we obtained transgenic mice expressing 1N4R P301S human tau under the murine prion promoter [57] from Jackson Laboratory, and maintained them on a B6/C3 background. The positive control mice were anesthetized at age 2.5 months with isoflurane and kept at 37 °C throughout the inoculation. We used 10 μL gas-tight Hamilton syringes to inject 10 µg of clone 9 cell protein lysate (previously described in [29, 47]) in the left hippocampus (bregma: -2.5 mm posterior, -2 mm lateral, -1.8 mm ventral). The mice were euthanized 4 weeks later as described below for seeding experiments.

### Mouse sample collection and preparation

The mice were anesthetized with isoflurane and perfused with chilled PBS + 0.03% heparin. Brains were post-fixed in 4% PFA overnight at 4 °C and placed in 30% sucrose in PBS until further use. Brains were sectioned at 50 μm with a freezing microtome and placed into cryoprotectant (32% ethylene glycol, 16% w/v sucrose, in 50 mM phosphate buffer pH 7.4, stored at -20 °C). 1 mm punches were then isolated from the left hippocampus using Miltex disposable punch biopsy tools. Four 4 mm punches were placed into 100 µl EDTA buffer (1 mM EDTA, pH 8.0), heated for 25 min at 95 °C and allowed to cool down for ~ 15 min. The samples were then sonicated with a water bath sonicator (Qsonica Q700MPX with chiller and tubing set) at 4 °C at 50 amplitude for 60 min, and stored at -80 °C until further use.

### Human autopsy samples

Human autopsy tissue used for this study was obtained from n = 20 individuals (10 females, 10 males, age range 50–93 years, Table 1) and 1 control (1 female, 30 years of age) in compliance with ethics committee guidelines at the University of Ulm as well as German federal and state law governing human tissue usage. Informed written consent for autopsy was obtained previously from the patients or their next of kin. Brains were fixed in a 4% buffered aqueous solution of formaldehyde for 14 days. Tissue blocks from 25 brain regions were excised and embedded in polyethylene glycol (PEG 1000, Merck, Carl Roth Ltd, Karlsruhe, Germany). 100 μm serial sections were collected and stained free-floating, as described previously [3] (Table 2). Brain tissue and the remaining tissue sections were stored for subsequent use in a 4% aqueous solution of formaldehyde at 8–15 °C for up to 26 years.

**Table 1:** Demographic and neuropathological data

Case	f/m	age	brain wt	NFT	Aβ	α-syn	TDP-43	APOE	Diagnoses
1	f	55	1455	I	0	0	−	ε3/4	intraventricular hemorrhage
2	m	55	1780	I	0	0	−	ε3/3	bronchopneumonia
3	f	50	1293	I	0	0	−	ε3/3	PCOM aneurysm, SAB
4	m	50	1600	I	0	0	−	ε4/4	M. Werlhof, hepatitis C
5	f	72	1070	I	3	0	−	ε3/4	cardiac failure
6	f	79	1165	I	0	0	−	ε3/3	coronary artery disease
7	f	93	1090	III (IV)	2	0	−	na	craniocerebral trauma
8	m	68	1380	III	0	0	−	na	aspiration pneumonia
9	m	72	1500	III	0	0	−	ε3/4	cardiac failure
10	m	74	1465	III	0	0	−	ε4/4	malignant neoplasm
11	m	76	1520	III	4	0	+	na	pneumonia
12	f	81	1130	III	2	0	−	ε3/3	acute myeloid leukemia
13	f	88	1495	III	3	0	−	ε3/4	cerebral hemorrhage parietooccipital
14	m	84	1170	V (VI)	3	0	+	ε3/4	AD
15	f	84	1175	V	3	2	+	ε3/3	AD, ILBD
16	m	58	1335	V	2	0	−	ε3/4	AD, myocardial infarction
17	f	72	1185	V	4	0	−	ε3/3	AD, craniocerebral trauma after falling
18	f	76	1405	V	4	0	−	ε3/4	AD, craniocerebral trauma after falling
19	m	76	1205	V	5	0	−	na	AD, cardiac failure
20	m	78	1460	V	5	0	−	ε3/4	AD
Control 21	f	30	1315	0	0	0	−	ε3/3	malignant neoplasm

20 cases fall into three NFT groups: NFT stage I (4 females, 2 males, 50–79 years); NFT stage III (3 females, 4 males, 68–93 years); NFT stage V (3 females, 4 males, 58–84 years). Abbreviations: **f, m**—female, male; **age**—age in years; **brain wt**—fresh brain weight in grams; **NFT**—Alzheimer's disease-related neurofibrillary tangle stage using Gallyas silver-iodide staining; **Aβ**—amyloid-β deposition phase using 4G8 IHC; **α-syn**—Parkinson disease-related neuropathological stage using α-synuclein IHC; **TDP-43**—43-kDa TAR DNA-binding protein neuronal inclusions; **APOE**—*APOE* allele status; **n/a** -not available; **AD**—Alzheimer's disease; **ILBD**—incidental Lewy body disease; **PCOM** -posterior communicating artery; **SAB**—subarachnoid bleeding

**Table 2:** Brain regions sampled

	Regions
1	Transentorhinal cortex (TRE)
2	Entorhinal cortex (EC, Brodmann Area 28)
3	Ammon's horn, sector 1 (CA1, hippocampal formation)
4	Amygdala, basolateral subnucleus (AMY)
5	Superior (first) temporal gyrus (STG, Brodmann Area 22)
6	Transverse temporal gyrus of Heschl (TTG, Brodmann Area 41)
7	Primary visual neocortex (PV, Brodmann Area 17)
8	Peristriate neocortex, high order sensory neocortex (Brodmann Area 19)
9	Anterior cingulate cortex—skeletomotor/emotion-autonomic integration (ACC, Brodmann Areas 24/32)
10	Retrosplenial/posterior cingulate cortex—memory/visuospatial orientation (RSC/PCC, Brodmann Areas 23/29/30)
11	Putamen (PUT)
12	Globus pallidus (GP)
13	Mediodorsal complex of thalamus (MD)
14	Orbitofrontal cortex (OFC, Brodmann Area 11)
15	Substantia nigra, pars compacta (SNpc)
16	Locus coeruleus (LC)
17	Basal nucleus of Meynert(BN)
18	Pontine gray (PG)
19	Inferior olivary nucleus (IO)
20	Cerebellar cortex (CC)
21	Cerebellar dentate nucleus (DN)
22	Internal capsule, anterior limb (IC)
23	Terminal stria (TS)
24	Olfactory bulb (OB)
25	Optic chiasm/tract (OC)

Punch biopsies were made from unstained sections of the 25 brain regions shown above using a 4 mm (3 mm for regions 19 and 22 in Experiment II) punch biopsy tool. Cross-contamination of seeding activity between individuals and regions was prevented by disposing the biopsy tool after each punch

### APOE genotyping

Apolipoprotein E status was available for 16/20 of the individuals studied (Table 1). The ε4 allele is a major genetic risk factor for sporadic AD [12], TDP-43 proteinopathy [55] and for dementia with Lewy bodies (DLB) and Parkinson’s disease dementia [9, 50, 53]. APOE genotyping was performed (E.G.) using a semi-nested polymerase chain reaction assay and restriction isotyping with restriction enzyme HhaI [21]. Genomic DNA was extracted from formaldehyde-fixed and paraffin-embedded brain specimens using the manufacturer’s protocols (QIAamp® DNA Mini Kit, Qiagen, Hilden, Germany).

## Neuropathological staging

Neuropathological staging and disease classification of AD-associated pathology were performed (H.B., K.D.T.) according to a previously published modified Gallyas silver-iodide staining protocol [3, 4] for recognition of phosphorylated somatic argyrophilic (fibrillary) neuropil threads (NTs) [1, 5] and neurofibrillary tangles (NFTs), as well as of extraneuronal ghost tangles (‘tombstone’ tangles) that display weak staining with the Gallyas method and strong staining with the Campbell-Switzer silver-pyridine method. In addition, AT8 immunohistochemistry (IHC, monoclonal anti-PHF-Tau antibody, 1:2000; Clone AT8; Pierce Biotechnology [Thermo Scientific] Waltham, MA [38], was performed. In contrast to the Gallyas method, AT8 IHC visualizes the broadest spectrum of intraneuronal pathological tau: argyrophilic NFTs of the Alzheimer type, NTs in dendritic processes, and non-argyrophilic axonal aggregates and pretangles. AT8 IHC detects ghost tangles less effectively than Gallyas silver staining, or not at all. The character and relative merits of thioflavin-S staining, Gallyas and Campbell-Switzer silver staining, as well as more conventional silver methods (the modified Bielschowsky and the Bodian methods) in relation to tau isoforms and to IHC have been discussed in detail elsewhere [51, 54]. We evaluated the presence of aging-related tau astrogliopathy (ARTAG) as proposed by Kovacs [32]. We visualized and staged Aβ deposition using the monoclonal anti-Aβ antibody 4G8 (1:5000; Clone 4G8; BioLegend, San Diego, CA) as recommended previously [26]. Clinical AD classification included cases with tau stages III/V and Aβ phases ≥ 2 [13, 15] (Table 1).

**Fig. 1:**
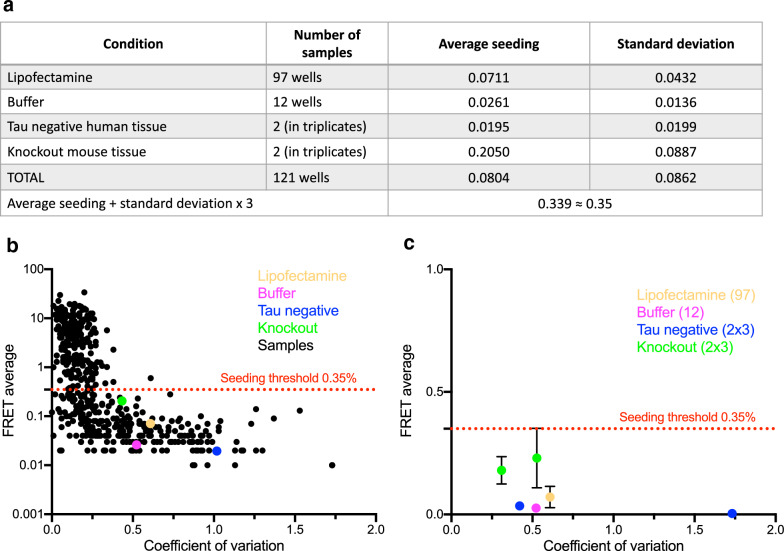
Seeding profile of cases (Experiment I). Punch biopsies were taken from 25 brain regions in 20 individuals (NFT stages I, III and V), homogenized, and transduced into v2H biosensor cells. Seeding was quantified by determining the percentage of FRET positive cells on a flow cytometer. Each sample was tested in biological/technical triplicate, and the average is reported. **a** Negative controls included cells that were treated with: (1) lipofectamine (+ buffer); (2) buffer only; (3) tau negative human brain tissue (from individual number 21 in Table 1; 4) brain tissue from tau knockout mice. The average seeding for each condition is shown as percentage of FRET positive cells ± standard deviation. We used the average of all negative samples and 3 × their respective standard deviations to determine the seeding threshold at 0.35% (in red). Only samples with seeding above 0.35% were scored positive. **b** The FRET average for each sample in experiment 1 was plotted on a log scale against the coefficient of variation (measure of assay precision defined as the standard deviation divided by the FRET average). The coefficient of variation is low for samples with seeding above the threshold of ~ 0.35%. For samples with seeding below this threshold, the coefficient of variation is significantly larger. **c** The FRET average for controls with standard deviation was plotted on a linear scale against the coefficient of variation. For lipofectamine and buffer controls, the average and standard deviation were derived from all 97 respectively 12 wells in this experiment. For tau negative human tissue and tau knockout mouse tissue, averages and standard deviation were calculated for each of 2 triplicates and plotted separately. Note that all controls ± standard deviation are below the seeding threshold of 0.35%. Color code: lipofectamine (yellow), buffer (pink), tau negative human brain tissue (blue), tau knockout mouse brain tissue (green), samples from individuals 1–20 (black)

**Fig. 2:**
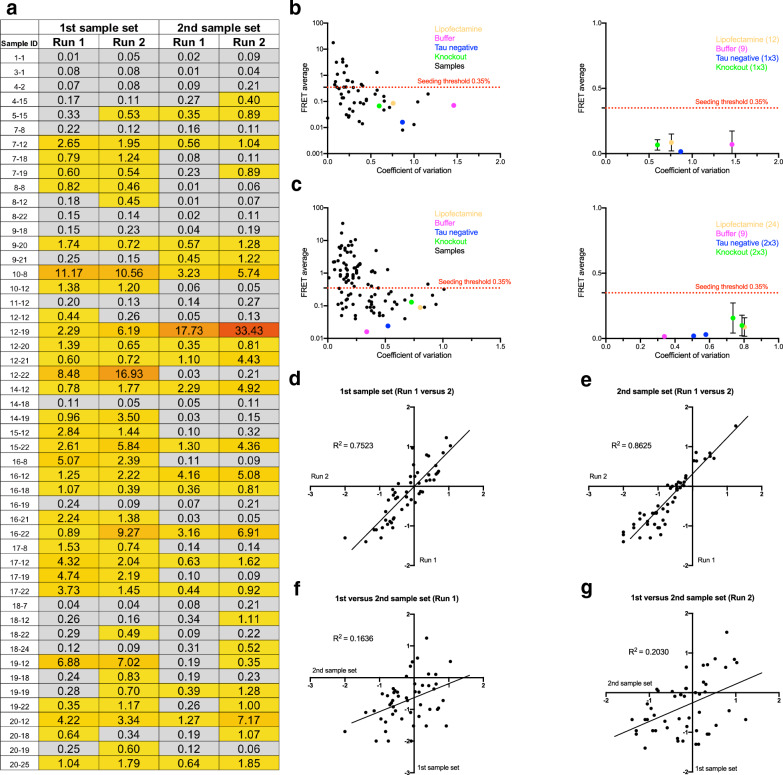
Reproducibility of seeding data. **a** To determine variation in seeding by region, we randomly selected a subset of 50 samples from the 1st sample set (tested in Exp I and shown in Fig. 1) and gathered a second set of punches from these brain regions (2nd sample set). Exp II tested seeding in the 2nd sample set (50 samples total plus controls). In Experiment III, seeding of both sample sets was tested a second time (100 samples total plus controls). In summary, both 1st and 2nd sample sets were tested in 2 separate runs, with the second run following a freeze–thaw cycle (Run 1 and 2). **b** and **c** show the plots for coefficient of variation versus FRET average for samples and controls in experiments II and III. The graphs were generated in the same way as for Experiment I in Fig. 1b and c. All control samples fall below the seeding threshold of 0.35%. Color code: lipofectamine (yellow), buffer (pink), tau negative human brain tissue (blue), tau knockout mouse brain tissue (green), samples from individuals 1–20 (black). **d** and **e** show a correlation analysis (linear regression) to test the reproducibility of seeding for the same sample sets between different experimental runs. **f** and **g** show the correlation analysis to test the reproducibility between different punches from the same brain region. Note that control samples were not included in graphs **d** to **g**

**Fig. 3:**
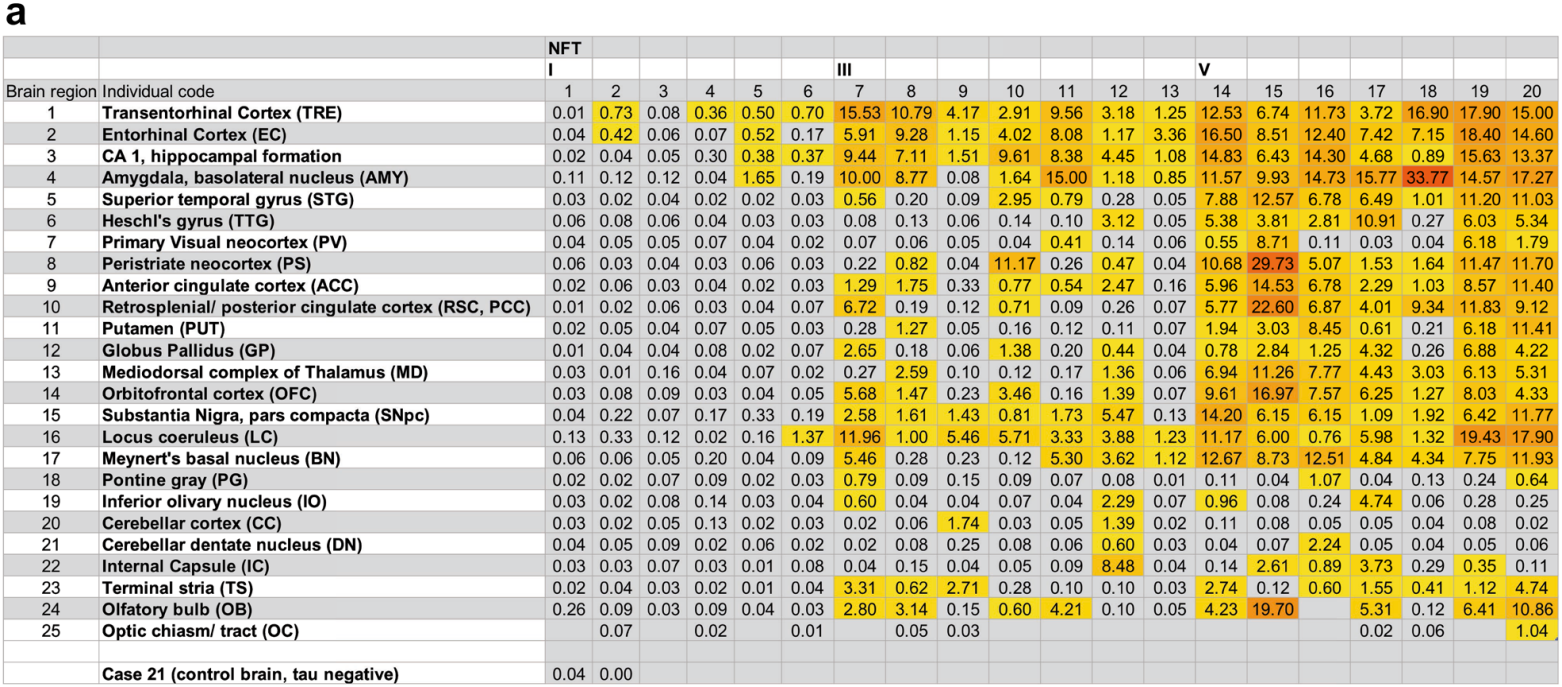

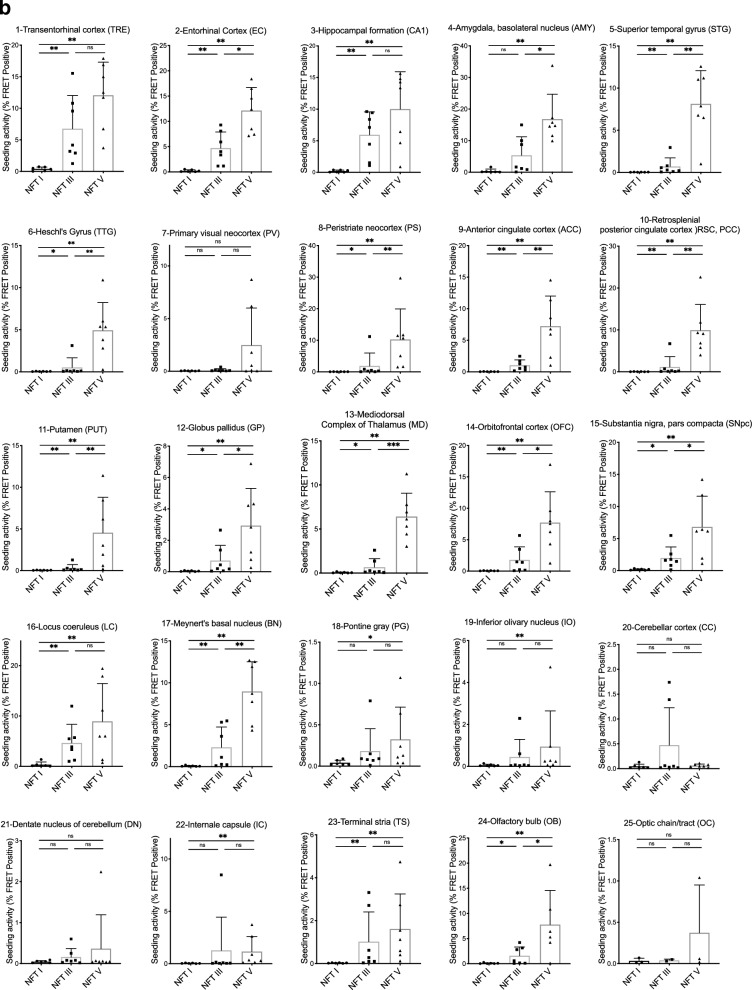
Seeding in 20 individuals, 25 brain regions (derived from Experiment I). **a** Seeding data heat map: Data points below the seeding threshold of 0.35% are colored in gray. Data points equal and above the seeding threshold are shaded with a graded color scale ranging from yellow (low) to red (high). Seeding data from human control brain (tau negative) was included as a comparison. **b** Seeding data from 25 brain regions plotted as individual graphs and separated according to NFT stage. Individual symbols (dot, square, triangle) represent data from individuals at each NFT stage. Statistical significance was determined by performing a non-parametric rank order test (Whitney-Mann test) to compare NFT I vs. III, III vs. V, and I vs. V. ns = non-significant, *p < 0.05, **p < 0.01. Errors bars show SD

**Fig. 4:**
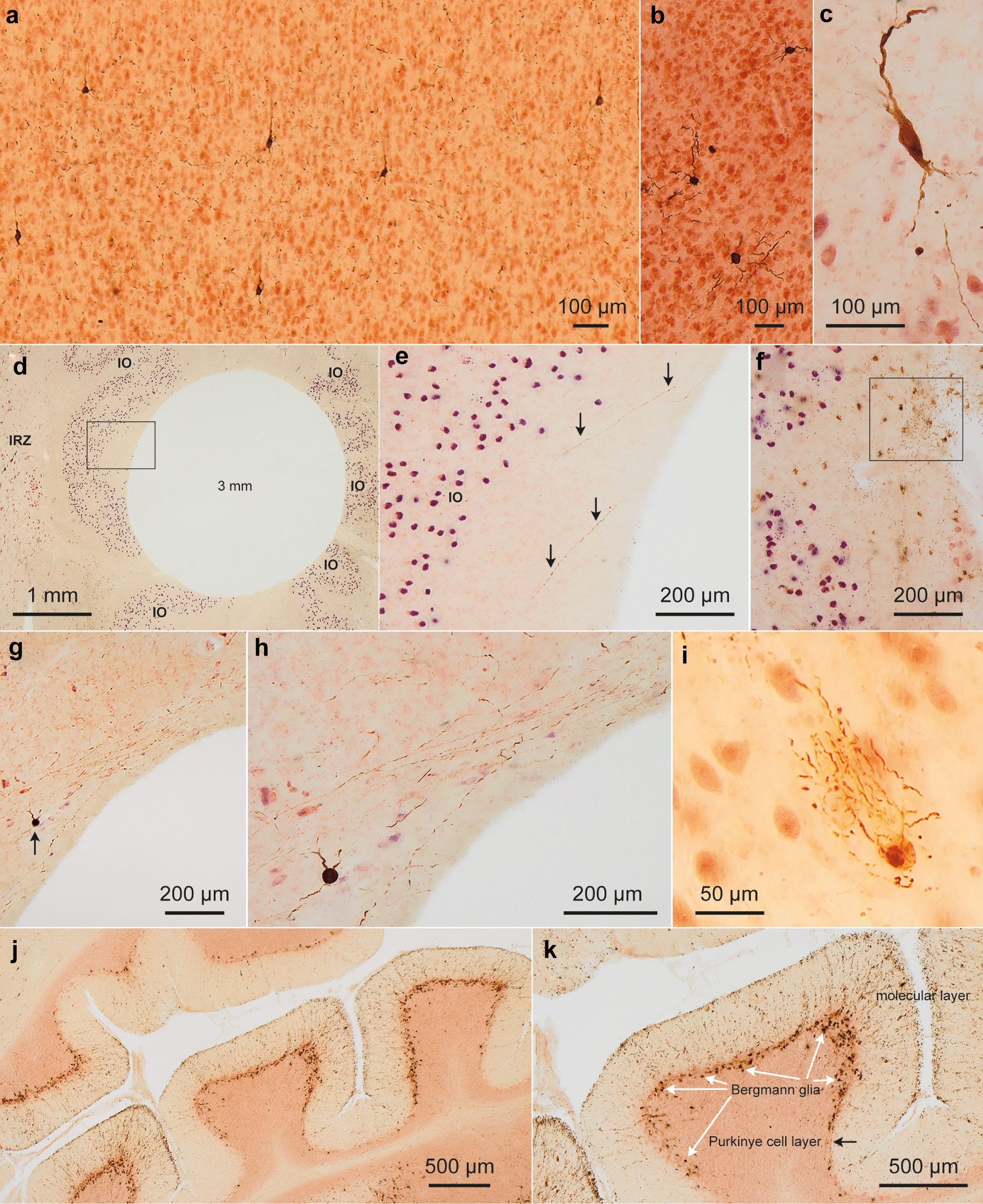
Phospho-tau Histopathology (Part I). **a** The TRE of case 1 (female, 55 years, NFT I, Table 1) displayed a few AT8-immunopositive neurons, but these were below the threshold for tau seeding (Fig. 3). With the exception of this case and case 3 (female, 50 years, NFT I, Table 1), the TRE of all 18 remaining individuals showed some degree of tau seeding activity (0.30–17.90, Fig. 3). **b** In case 16 (male, 58 years, NFT V, Table 1), moderate AT8 pathology (neuronal somata, axons) and low tau seeding (1.07) were detectable in the PG. **c** Punches mistakenly located in the dorsal raphe nucleus (DRN) of one individual (case 7, female, 93 years, NFT III, Table 1) showed AT8-immunoreactive neuronal pathology combined with mild tau seeding (0.79). **d** Some IO punches (case 7), with no AT8-positive cell bodies, contained isolated AT8-positive axons and low tau seeding activity (0.60). **e** Framed area in **d** at higher magnification. *Arrows* indicate two AT8-positive axons. **f** In the IO of case 7 (same individual as in **c-e** and **g, h**) we noted unexpected and marked aging-related tau astrogliopathy, ARTAG (example in the framed area). **g, h** A single AT8-positive neuron was seen in this punch from the GP accompanied by numerous AT8-immunoreactive axons (see also Fig. 5f), the latter possibly originating in the basal nucleus of Meynert (BN). Tau seeding in the GP of this case was moderate (2.65); notably, the BN of this individual also featured moderate tau seeding (5.46). **I.** The DN of case 9 (male, 72 years, NFT III, Table 1) had few AT8-positive cell bodies (pretangles) and was sub-threshold for tau seeding (0.25). In cases at NFT stages I and III, only one (case 12, female, 81 years, NFT III, Table 1) had mild tau seeding in this region (0.60). **j, k** Notably, in the CC of case 12 (female, 81 years, NFT III, Table 1), the Bergmann glia (here, as thorn-shaped astrocytes) and, in the molecular layer, their astrocytic processes were AT8-immunopositive and also had tau seeding (1.39). By contrast, the neurons in the Purkinje layer were AT8-negative and negative for tau seeding. Tau seeding (1.74) was detected in the CC of one additional individual (case 9, male, 72 years, NFT III, Table 1, Fig. 3)

**Fig. 5:**
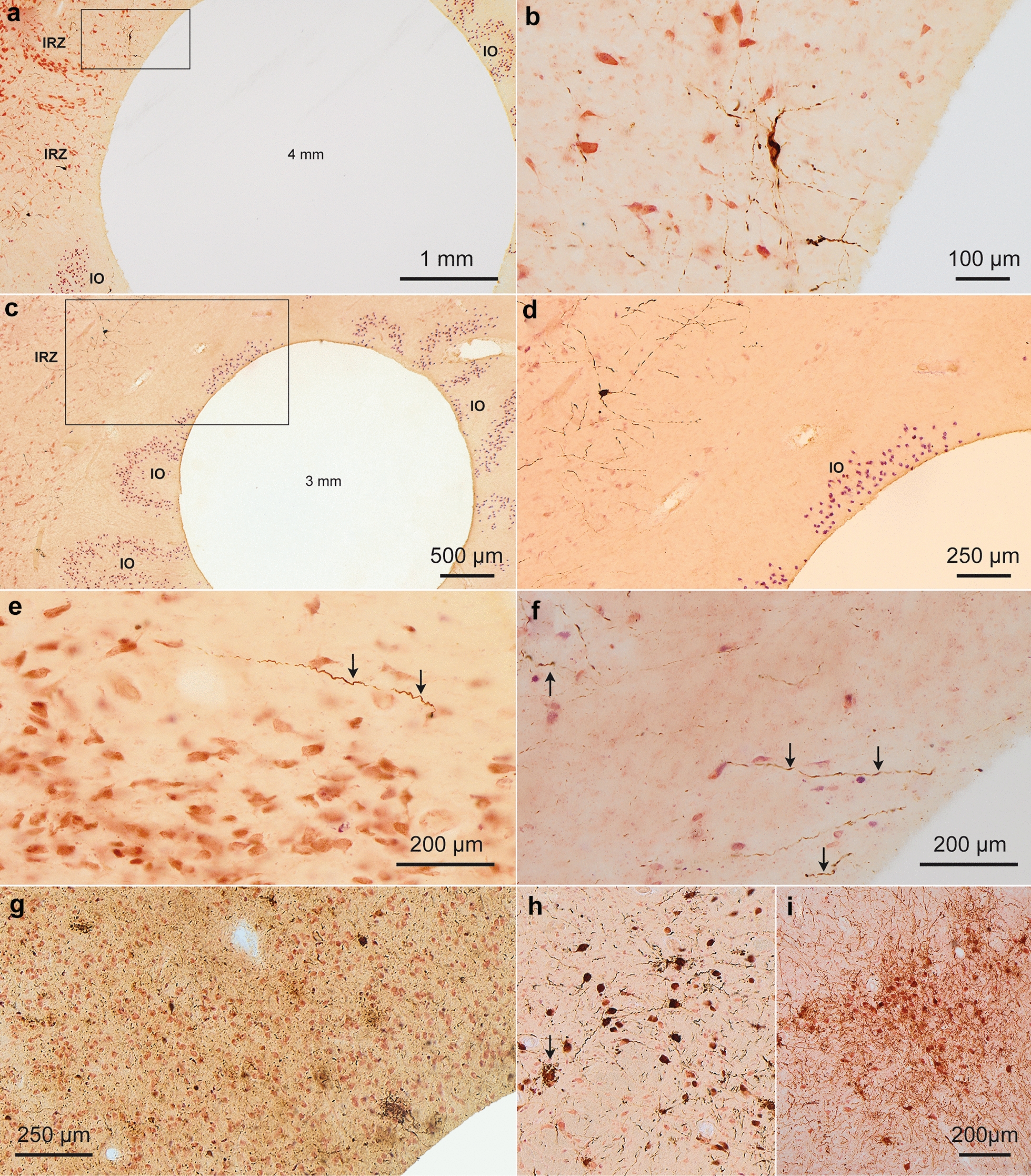
Phospho-tau Histopathology (Part II). **a** A 4 mm punch from the IO of case 16 (male, 58 years, NFT V) included portions of the immediately adjacent intermediate zone (IRZ) with strongly AT8-positive neurons and axons. In the IO itself, a single AT8-immunoreactive axon was detectable and some perivascular ARTAG was present, but none of the neurons there were AT8-immunoreactive. Tau seeding was below threshold (0.24). In at least two additional individuals (cases 12 and 17), prominent AT8-positive neurons and axons in the IRZ accompanied by IO AT8-negative neurons as well as perivascular ARTAG may have accounted for the tau seeding signals in IO punches (2.29 and 4.74). **b** Framed area in **a** at higher magnification showing AT8-positive tau pathology at the punch edge. **c** A 3 mm punch with edges free of AT8 pathology from case 15 (female, 84 years, NFT V), in which no portions of the IRZ were included. **d** Framed area in **c** at higher magnification displaying a clean punch edge and, directly beyond it, AT8-positive pathology in the IRZ. Tau seeding activity in the IO of this case was below threshold (0.08). **e** AT8-positive axon (*arrows*) in the substantia nigra, pars compacta (SNpc) of case 13 (female, 88 years, NFT III). Some tau seeding in the SNpc was present in 13/20 individuals (0.81–14.20), and was exceeded in the lower brainstem only by 15/20 cases in the LC (1.00–19.43). **f** *Arrows* point to AT8-positive axons in the GP of case 7 (female, 93 years, NFT III) (see also Fig. 4g, h). **g** The highest propensity for tau seeding (33.77) was detected in the basolateral subnucleus of the amygdala (AMY) of case 18 (female, 76 years, Stage V), where severe diffuse neuronal AT8-pathology was accompanied by some AT8-immunopositive astrocytes (ARTAG). **h, i** Tau seeding activity in the LC first became more pronounced during NFT stages III and V, e.g., 11.96 in case 7 (female, 93 years, NFT III) (**h**); 19.43 in case 19 (male, 76 years, NFT III); and 5.71 in case 10 (male, 74 years, NFT III) in micrograph **i**. *Arrow* in **i** points to extraneuronal neuromelanin lying free in the neuropil after severe neuronal loss

We excluded other non-AD tauopathies, including argyrophilic grain disease, progressive supranuclear palsy, Pick’s disease, corticobasal degeneration, and Niemann-Pick disease type C. Separate sets of 100 µm free-floating sections from all cases were immunostained using the following primary antibodies: (1) a monoclonal anti-syn-1 antibody (1:2000; Clone number 42; BD Biosciences, Eysins, Switzerland) for detection of Lewy body disease-related α-synuclein inclusions [26]; (2) a polyclonal rabbit antibody recognizing the N-terminal of normal TDP-43 (1:5000; Proteintech, Manchester, UK) [52]. We staged all cases for sporadic Parkinson’s disease (PD), as described elsewhere [8] (Table 1). One case showed incidental α-synuclein-positive Lewy pathology; three cases displayed coincident TDP-43 immunoreactivity [17, 34] (Table 1). Histological slides were viewed with an Olympus BX61 microscope (Olympus Optical, Tokyo, Japan). Pathology was assessed semi-quantitatively on a four-point scale: 0 = no detectable tau inclusions, +  = mild (at least one or two AT8-positive cell soma/somata); +  +  = moderate inclusions; +  +  +  = severe inclusions. Digital micrographs of IHC-stained sections (Figs. 4, 5) were taken with an Olympus XC50 camera (H.B.) using the Cell D® Imaging Software (Olympus, Münster, Germany). The extended focal imaging (EFI) function was used for stacking images at different optical planes (Cell D Imaging Software, Olympus, Münster, Germany). The EFI algorithm extracts the image features with the sharpest contrast from all layers of the stack and merges them into a single image.

### Punch samples

From each case, including the negative human control, punch samples were collected (K.D.T.) free-floating from unstained sections of the 25 brain regions shown in Table 2 with a punch biopsy tool (Kai Industries Co, Ltd. Japan) – with diameter of either 4 mm (resulting in a punch volume of ~ 1.257 mm^2^) or 3 mm (with estimated punch volume of ~ 0.706 mm^2^). The 3 mm punch device was only used for the internal capsule (IC) and the inferior olivary nucleus (IO) in the 2nd set of punches to ensure that the punches were confined to the immediate target regions. To avoid cross contamination of seeding between individuals and regions, punch tools were used only once. Samples were encoded and all subsequent preparation and seeding assays were performed in a blinded fashion. Tissue punches were stored in 1 × TBS at 4 °C until use. Brain tissue was collected in the same way with the 4 mm punch tool from positive and negative control mice (S.E.).

### Human sample preparation

One 4 mm punch or two 3 mm punches were placed into 100 µl EDTA buffer (1 mM EDTA, pH 8.0), heated for 25 min at 95 °C and allowed to cool down at 4 °C for ~ 15 min. The samples were then sonicated with a water bath sonicator (Qsonica Q700MPX with chiller and tubing set) at 4 °C at 50 amplitude for 60 min, and stored at −80 °C until further use.

### Transduction of biosensor cell lines, flow cytometry and seeding analysis

The seeding assay was conducted as previously described with the following changes: biosensor cells were plated at a density of 25,000 cells/well in a 96-well plate in a media volume of 130 µl per well. The mouse and human tissue samples were thawed on ice, followed by thorough vortexing and incubation with Lipofectamine 2000 for 30 min. 1 µl of tissue lysate with 0.5 µl of lipofectamine and 18.5 µl of OptiMEM (Gibco, Life Technologies) was added to each well, resulting in 20 µl total. For each experiment, negative controls received either Lipofectamine in OptiMEM (lipofectamine controls), or OptiMEM (buffer controls). The lysate-lipofectamine mix was applied to the cells, and cells were incubated for an additional 72 h. Cells were harvested with 0.05% trypsin and fixed in 2% PFA for 10 min, then resuspended in flow cytometry buffer (HBSS plus 1% FBS and 1 mM EDTA). An LSRFortessa SORP (BD Biosciences) was used to perform FRET flow cytometry. We quantified FRET as previously described with the following modification: we identified single cells that were double-positive for mCerulean and mClover and subsequently quantified FRET positive cells within this population. For each data set, 3 technical replicates were included. Data analysis was performed using FlowJo v10 software (Treestar Inc.), GraphPad Prism v8.4.3 for Mac OS X, and Excel v16.16.25 (Microsoft).

### Statistical analyses

Samples were collected at the University of Ulm and cases were blinded prior to seeding analyses by B.E.S. at UT Southwestern Medical Center. Flow cytometry gating and analysis of seeding were completed prior to the decoding and interpretation of the seeding results. All statistical analysis was performed using GraphPad Prism v8.4.3 for Mac OS X and Excel v16.16.25 (Microsoft). Statistical significance between seeding at different NFT stages was determined by performing a non-parametric rank order test (Whitney-Mann test). Correlation analysis (linear regression) was performed and Spearman r correlation was calculated to test the reproducibility of seeding between different experimental runs.

### Generation of 3D tau seeding map

Magnetic resonance imaging of the brain was performed in a healthy volunteer using a 3 T Siemens Prisma MRI scanner, including acquisition of 3D T1-weighted MPRAGE sequence with 1 mm^3^ isotropic resolution. Segmentation of regions of interest (ROIs) within the brain was performed semi-automatically using FreeSurfer (version 5.3.0, http://surfer.nmr.mgh.harvard.edu). In cases where ROIs did not already exist in the FreeSurfer library, manual segmentation was performed by an experienced board-certified neuroradiologist (F.F.Y.) using the Segmentation Editor tool in 3D Slicer (version 4.10.2, https://www.slicer.org/). The weighted average tau deposition from all subjects within each NFT stage group was then applied to each ROI using a customized script in Matlab (version R2015b). Brain regions were included only if at least one subject within each NFT stage exhibited seeding. The Build Surface function within Mango (version 4.1, http://ric.uthscsa.edu/mango/) was then used to visualize the ROIs within a 3D projection of a control brain.

## Results

### Sampling of 25 brain regions across 20 individuals

Previous publications from our group and others have studied seeding in a limited number of brain samples from AD patients using the biosensor system [14, 20, 25, 28]. We chose 20 individuals with confirmed AD pathology for this analysis (Table 1). Given that the differences in tau tangle pathology between NFT stage I/II, III/IV, and V/VI are subtle, we limited our study to NFT stages I, III and V. Furthermore, we used the punch device established by Kaufman et al. 2018 [28] for more precise sampling of the 25 brain regions of interest as opposed to sampling by dissecting large tissue pieces (Table 2).

### Seeding threshold determination

To determine the lower limit of detection, tissue lysate was transduced into v2H biosensor cells. We quantified the percentage of FRET positive cells on the flow cytometer as a correlate of intracellular tau seeding, compared to negative control samples. Negative samples included human tau-negative brain tissue from case 21 (taken from the pons), brain tissue from tau knockout mice, and wells treated with lipofectamine or buffer only. Note that the samples from case 21 were included as internal assay control to ensure that the seeding assay was appropriately detecting tau seeding only when present and not detecting seeding when tau was absent in human tissue. Therefore, we did not sample each of the 25 brain regions from case 21. We used the average of all negative samples plus 3 standard deviations to determine the “positive” seeding threshold. We considered samples with seeding of 0.35% and above to be positive, whereas samples below this threshold were considered negative. With a 3 SD cutoff, we increased the specificity of our analysis at the cost of sensitivity. The coefficient of variation (defined as standard deviation divided by the FRET average) was used as a measure of precision and is high for samples below the seeding threshold and low for samples above the threshold (Fig. 1). Given the relatively high specificity of the assay, negative results do not rule out tau seeding in a given sample.

### Reproducibility between experimental runs and samples

To test seeding within regions, we selected 50 punches from 20 individuals and obtained a second set of punches from the same section as the 1st set whenever possible. If not possible (because of limited amount of tissue), the 2nd punch was obtained from an immediately adjacent section. We then performed 2 additional seeding experiments: In experiment II, the 2nd sample set was tested (run 1). In experiment III, both sample sets were thawed a second time and tested for seeding (run 2). Thus, the 1st sample set was tested after one freeze thaw cycle in experiment I (run 1), and after a second freeze–thaw cycle in experiment III (run 2). In the same way, the 2nd sample set was tested in experiment II (run 1) and experiment III (run 2) after one versus two freeze–thaw cycles (Fig. 2). For both experiments II and III, the coefficient of variation above the previously defined seeding threshold of 0.35% was low (Fig. 2b and c). We then used linear regression to compare the sample sets and experimental runs. Seeding correlated well between different experimental runs (run 1 and 2) of the same samples with R^2^ in the range of 0.7 – 0.8 (Fig. 2d, e). The reproducibility of the seeding data between different samples (1st versus 2nd sample set) had low reliability (Fig. 2f, g).

### Progressive accumulation of seeding within individuals

To examine the progression of seeding across all brain regions, we created a heat map with the seeding for each individual and each brain region (Fig. 3a). We also plotted seeding for each individual brain region for all 20 individuals (Fig. 3b) and generated a 3D seeding map for each NFT stage (Additional file 2: Figure S6). In general, seeding increased with higher NFT stages in all brain regions examined. For unexpected seeding results (Table 3), we performed AT8 staining on selected brain regions to test for tau deposition (Additional file 1: Table S1).

**Table 3:** Unexpected seeding results

Brain region	Expectation	Reference for expectation	Findings
1-TRE	Seeding in all cases	Kaufmann et al. [28]	Unexpected = > n = 2/6 cases at NFT stage I did not show seeding
4-AMY	Seeding starting at late NFT stages	Braak et al. [4]	Unexpected = > seeding in n = 1/6 NFT stage I case, in n = 6/7 NFT stage III cases and in all NFT stage V cases
6-TTG	Seeding starting at late NFT stages (V-VI)	Braak et al. [4]	Unexpected = > seeding in n = 1/7 NFT stage III case; expectedly in n = 6/7 NFT stage V cases
12-GP	Seeding not expected since it typically does not develop tau pathology (in contrast to Aβ plaques) in AD	Braak and Del Tredic [6]	Unexpected = > seeding in n = 3/7 NFT stage III cases and in n = 6/7 NFT stage V cases
15-SN	Seeding starting at late NFT stages (V-VI)	Braak et al. [4]	Unexpected = > seeding seen earlier—in most NFT stage III cases (n = 6/7) and in all NFT stage V cases
18-PG	Seeding not expected	Braak et al. [4]	Unexpected = > seeding in n = 1/7 NFT stage III case and in n = 2/7 NFT stage V cases Note: The dorsal raphe nucleus (DRN) in case 7 was punched accidentally and likely explains the seeding in this sample
19-IO	Seeding not expected	Braak et al. [4]	Unexpected = > seeding in n = 2/7 NFT stage III cases and in n = 2/7 NFT stage V cases Note: Some punches from IO were contaminated by overlapping AT8-positive neurons/axons in the IRZ
20-CC	Seeding not expected	Braak et al. [4]	Unexpected = > seeding in n = 2/7 NFT stage III cases
21-DN	Seeding not expected	Braak et al. [4]	Unexpected = > seeding in n = 1/7 NFT stage III case and in n = 1/7 NFT stage V case
22-IC	Seeding not expected	Braak et al. [4]	Unexpected = > seeding in n = 1/7 NFT stage III case and in n = 4/7 NFT stage V cases
24-OB	Seeding starting at early/intermediate NFT stages (II-III)	Attems et al. [2]	As expected, seeding in n = 4/7 NFT stage III cases; however, unexpected = > in only n = 5/6 NFT stage V cases
25-OC	Seeding expected	DeVos et al. [14]	Unexpected = > seeding in only one individual (case 20)

Summary of seeding results that were unexpected based on seeding results and/or neuropathology data from prior studies. For a complete list of all brain regions and expectations prior to the study refer to Additional file 1: Table S2. For unexpected results, AT8 IHC was performed as listed in Additional file 1: Table S1

### Tau seeding starts in the transentorhinal cortex

We detected seeding in the transentorhinal cortex (TRE) in all individuals at NFT stages III and V (Fig. 3a). At NFT stage I, the majority of individuals (n = 4/6) had positive seeding in the TRE. In 2 cases (cases 1 and 3), seeding in the TRE was below the threshold of detection despite the presence of 9 and 1 AT8-positive neuron(s) respectively in this region (Additional file 1: Table S1 and Fig. 4a).

### Early seeding in the entorhinal cortex, CA1, amygdala, and locus coeruleus

Four additional brains regions demonstrated early seeding. The entorhinal cortex (EC) and CA1/hippocampal formation scored positive in n = 2/6 NFT stage I cases, and remained positive at all higher NFT stages (n = 14/14) (Fig. 3a). Seeding was also detected in n = 1/6 NFT stage I individuals in the basolateral subnucleus of the amygdala (AMY), and this region remained positive in the vast majority of NFT III (n = 6/7) and all NFT V cases (n = 7/7) (Figs. 3a and 5g). The locus coeruleus (LC) exhibited seeding in n = 1/6 at NFT stage I, and at NFT stages III and V the LC was positive for seeding in all individuals tested, 2 of whom displayed strong seeding at NFT stage V (Figs. 3a and 5h, i). This is broadly consistent with our prior work [28].

### Intermediate seeding in 9 brain regions

The superior temporal gyrus (STG), the peristriate neocortex (area 19, PS), and the terminal stria (TS) scored positive for seeding in some individuals (n = 3/7) at NFT stage III, and in all individuals at NFT stage V (Fig. 3a). The anterior cingulate cortex (ACC) demonstrated positive seeding in most individuals at NFT stage III (n = 5/7) and all NFT stage V cases, and the orbitofrontal cortex (OFC) as well as Meynert’s basal nucleus (BN) were seeding-positive in n = 4/6 cases at NFT stage III and in all individuals at NFT stage V (Fig. 3a).

We detected robust seeding in the substantia nigra, pars compacta (SNpc) in most cases (n = 6/7) at NFT stage III and n = 7/7 at NFT stage V (Fig. 3a). By contrast, tangle pathology is typically observed in this region only at later stages (NFT V and VI) [4]. AT8 staining in n = 5 seeding-positive cases (e.g., at NFT stages I and III, Additional file 1: Table S1) revealed immunopositive SNpc axons in all individuals examined (Fig. 5e). However, seeding above threshold could only be detected in one case (case 12) that also displayed AT8-positive neurons (Additional file 1: Table S1).

The globus pallidus (GP) does not typically show AD-associated tau pathology [7]. However, n = 4/7 cases at NFT stage III and all cases at NFT stage V demonstrated seeding (Fig. 3). AT8 staining in 9 individuals revealed AT8 positivity in axons but not nerve cell somata (Additional file 1: Table S1). In two separate punches from case 7 (Fig. 4g and h), we found a single AT8-positive neuronal body in the GP. Seeding-positive axons in the GP could have their origins in the basal nucleus of Meynert (BN), which displayed seeding in n = 4/7 cases at NFT stage III and in n = 7/7 NFT stage V cases (Fig. 3).

The olfactory bulb (OB) scored positive in n = 9/19 cases (Fig. 3a). We found no evidence of tau seeding at NFT stage I (Fig. 3a). At NFT stage III, n = 4/7 individuals displayed seeding in this region, and at NFT stage V, n = 5/6 individuals scored positive, 1 of which was particularly pronounced (case 15). No OB tissue from case 16 was available.

### Late seeding in 5 brain regions

Seeding at late NFT stages was present in 5 brain regions. Heschl’s gyrus (TTG), the putamen (PUT), retrosplenial/posterior cingulate cortex (RSC/PCC) and mediodorsal complex of thalamus (MD) were positive for seeding in only n = 1/7 to n = 2/7 individuals at NFT stage III, and in most or all individuals at NFT stage V (Fig. 3). The primary visual neocortex (PV) had seeding in only n = 1/7 individuals at NFT stage III, and at NFT stage V n = 4/7 individuals scored positive.

### Occasional/inconsistent seeding in 6 brain regions

We observed seeding and AT8-positive tau pathology in some individuals at higher NFT stages (III and V) in 6 additional brain regions, which is a new finding in some cases.

#### Pontine gray

Three individuals (n = 1 at NFT stage III and n = 2 at NFT stage V) scored positive for seeding in the pontine gray (PG). Of note, for case 7 (with positive seeding of 0.79 in the PG), the dorsal raphe nucleus (DRN) was punched accidentally instead of PG for the seeding assay (Fig. 4c). AT8 staining in n = 8/20 cases showed (mostly mild) AT8 changes in neurons and neurites/axons of the PG in n = 4 cases, of which n = 1 (case 16) had seeding (Fig. 4b).

#### Inferior olivary nucleus

4 individuals (2 at NFT stage III and 2 at NFT stage V) had seeding in the inferior olivary nucleus (IO). AT8 staining in 9 cases was negative in neurons. However, three cases had tau-positive astrocytes consistent with aging-related astrogliopathology (ARTAG) (Fig. 4f), and 6 cases had AT8-positive axons (Fig. 4d and e). Note that obtaining punches from the IO is technically challenging because of its close proximity to the intermediate reticular zone (IRZ) (Figs. 4d, e and 5a-d), which can contain AT8-positive axons and/or neurons in early AD stages [46]. While we obtained mostly clean punches from IO, we cannot exclude that a few contained edges of the IRZ, as noted in Additional file 1: Table S1 and shown in Fig. 5a and b. Use of a 3 mm punch helped to minimize such occurrences.

#### Cerebellar dentate nucleus and cerebellar cortex

In two individuals, the cerebellar dentate nucleus (DN) and/or cerebellar cortex (CC) had seeding (Fig. 3a). Interestingly, AT8 staining for the two positive CC regions (cases 9 and 12) revealed AT8-positivity in the Bergmann glia (as thorn-shaped astrocytes) and in their astrocytic processes (Fig. 4j and k) rather than in neurons, which is a novel ARTAG finding. In the DN of 4 cases, nerve cells plus axons were AT8-positive (Fig. 4i).

#### Internal capsule, anterior limb

We observed seeding in the anterior limb of the internal capsule in n = 1/7 case at NFT stage III, and in n = 4/7 cases at NFT stage V (Fig. 3a). AT8 staining in 5 cases revealed positive axonal signals. The anterior portion of the inner capsule contains both axons of the frontopontine projection and axons that originate in the mediodorsal complex of thalamus (MD) [42]. Neither the IC nor the OFC or MD were above threshold for seeding in NFT stage I, but this shifted at NFT stage III, and OFC and MD displayed strong seeding at NFT stage V.

#### Optic chiasm/tract

Only n = 1/8 individual at NFT stage V (case 20) showed positive seeding in the optic chiasm/tract (OC) (Fig. 3a). Additional tissue from this brain region was not available to obtain reliable AT8 staining. Seeding in the OC might originate in axons from the DRN, the lower raphe nuclei, LC, amygdala, EC, superior colliculus, and/or the retina [42, 49].

### ApoE status does not influence seeding in our study

ApoE genotyping was available for n = 16/20 cases. 8 carried at least one ApoE ε4 allele, and 2 were homozygous for ApoE ε4 (Table 1). We detected no significant influence of ApoE on the seeding in all cases comparing E4 carriers versus non-carriers.

### Other tauopathies can be excluded in our cohort

None of the cases displayed the tauopathy changes specific for CTE [37] or PSP [16, 33, 43, 45].

## Discussion

We used an ultrasensitive biosensor assay to create a comprehensive map of seeding in the AD brain, analyzing 25 brain regions (15 of which had not been analyzed previously) across 20 individuals with AD pathology at NFT stages I, III, and V. We used a punch biopsy for precise sampling of regions of interest, and defined the positive seeding at 3 SD above the average of all negative samples tested, a threshold with relatively high specificity and low sensitivity.

### Variability within brain regions

We observed variability between different punches from the same brain regions, highlighting the importance in future studies of sampling larger brain volumes or averaging multiple biopsies. We used small punches with a diameter of 3–4 mm to increase sampling precision, especially in closely adjacent anatomical regions, such as the IO and IRZ. However, smaller diameter punches also increased the variance in tau seeding detected, especially in areas with scarce tau pathology, e.g., the IC. Further, given the small size of some brain regions examined we occasionally sampled adjacent sections, which may have contributed to the variability. Finally, the high threshold to register a positive signal may have contributed to the variability in regions with low numbers of tau-positive neurons. In prior work [28], adjacent punches from the TRE/EC correlated relatively well, which may be due to its more easily defined structure, or more homogeneously distributed pathology than many of the brain regions studied here.

### Seeding increases with neuropathological stage

Some brain regions studied are not known to develop tau pathology in AD, yet we observed tau seeding across all 25. Many regions included in this study have not previously been studied for tau seeding. Our predictions regarding seeding were based on prior neuropathological data and our knowledge of the neuroanatomical connections that determine input and output for these brain regions. Our predictions were accurate in many cases (Additional file 1: Table S2), e.g., as predicted, we found early seeding in the transentorhinal and entorhinal cortex; seeding at intermediate stages in the superior temporal gyrus (STG) and the anterior cingulate cortex (ACC); and seeding at intermediate/late stages in the putamen and the mediodorsal complex of thalamus (MD). These findings were consistent with the tau progression pathway described in prior studies [4, 14, 28].

### Tau seeding starts in the TRE

The majority of individuals tested in our study scored positive in the TRE, excepting 2 individuals at NFT stage 1 (cases 1 and 3). Interestingly, TRE sections of both individuals 1 and 3 contained up to 9 AT8-positive neurons and scored negative for seeding, possibly due to the low density of AT8-positive neurons and the relatively high seeding threshold we used (Additional file 1: Table S1). In other words, the number of AT8-positive structures (i.e., “severity” of pathology) in the TRE may not account for the level of seed-competent tau. Prior work from our group indicates that early tau pathology starts in the TRE/EC region [28]. Of note, Kaufman et al. [28] did not separate the TRE and EC, as we did here. The differences in seeding between TRE and EC were subtle: compared to the EC, the TRE had seeding in more individuals (n = 4 vs. n = 2) at NFT stage I, and the average seeding was slightly higher at NFT stage I. However, the slightly earlier involvement of TRE is consistent with its classification as NFT stage I [4].

### Tau seeding precedes tau pathology

Some brain regions show seeding significantly earlier than predicted based on neuropathological staging (SNpc, AMY, TTG/Heschl’s gyrus). This confirms prior observations that tau seeding can precede the development of tau pathology as detected by immunohistochemistry [14, 20]. Work from our laboratory has shown that the seeding assay reliably detects monomeric and oligomeric tau seeds [39, 40], and we speculate that the presence of seed-competent tau monomer and oligomers can precede the presence of AT8-positive tangles. Studies in the PS19 mouse model support these conclusions [25].

### Tau seeding occurs in unanticipated brain regions

We detected tau seeding in brain regions not known to develop tau pathology, i.e. the GP, IC, PG, IO, and cerebellum. The seeding in these brain regions was low overall, and neuropathological findings/AT8 staining and seeding did not necessarily correlate with each other. In some regions, subsequent AT8 staining revealed the presence of tau predominantly in astrocytes (see below). We also detected tau seeding in regions not known to exhibit tau seeds, although observed to develop neurofibrillary pathology during AD. These included the IRZ, DRN, AMY, BN, and OB. The seeding was not always consistent between connected anatomical regions within each individual case, since punches from different regions were taken randomly from the right and left hemisphere, and we did not intend to analyze direct anatomical connectivity between specific brain punches. Therefore, we cannot speculate about anatomical pathways that could explain seeding in these samples. However, we note that the AT8-positive axons observed in the GP could belong to the BN. Seeding levels in the BN in all such cases were always comparatively higher than in their respective GP punches (Fig. 3a).

### Aging-related tau astrogliopathy (ARTAG)

In some brain regions with unexpected presence of tau seeding (globus pallidus, inferior olivary nucleus, cerebellar cortex), we detected tau pathology in astrocytes or in Bergmann glia. It is not known how abnormal tau in astrocytes accumulates [18]. ARTAG develops mainly, but not exclusively, in individuals over 60 years of age. The two major cytomorphologies are thorn-shaped astrocytes (TSAs) and granular or fuzzy tau immunoreactivity in astrocytic processes (GFA). TSAs occur in subpial, subependymal, or perivascular areas, as well as white matter [31, 32]. It is now generally accepted that astroglia either have no endogenous tau [22] or, very low levels [31]. It is unclear why tau accumulates in astrocytes, and whether it transfers between them. We hypothesize that they phagocytize it from the surrounding interstitial space, while tau seeds might originate in neurons [18]. Ferrer et al. have described seeding in AT8-positive astrocytes in cases displaying minimal intraneuronal tau pathology without determining where seed competent tau originates [18]. The pathophysiological significance of ARTAG for AD is unknown [31] and will require further studies. Our prior studies of tau strains indicate that some preferentially involve astrocytes [29, 47].

### Tau seeding in the cerebellum and tau pathology in Bergmann glia

We predicted that the cerebellar dentate nucleus (DN) and the cerebellar cortex (CC) would not exhibit seeding, however, for each region, n = 2/20 cases did. We did not observe AT8-positive tau pathology in the somatodendritic or axonal compartments of cerebellar neurons, e.g., granule cells, Purkinje cells, or stellate/basket cells. Notably, AT8 staining for the 2 seeding-positive CC regions revealed AT8 staining of Bergmann glia rather than neurons and axons (Fig. 4j, k) (see remarks under the previous heading ARTAG). We previously observed low tau seeding in the cerebellum at late stages [20] but tau pathology in Bergmann glia has not been described. The findings raise the possibility of a sub-type of AD, with a tau strain that preferentially affects Bergmann glia cells.

### Summary and conclusion

In the future, we will focus on selected brain regions with notable and/or new results, such as the IRZ, AMY, BN, IC, GP, cerebellum, and OB. Moreover, an analysis of tau strains in all of the regions studied here and in the hypothalamus [4] would be informative. Importantly, although our data are limited by the number of individuals analyzed (n = 6–7 per NFT stage), we have observed tau seeding in regions where AT8 staining is negative. This suggests that tau pathology extends beyond the brain regions that are routinely included in AD staging, and is highly variable between individuals. Thus, immunohistochemistry and seeding are two fundamentally different and yet complementary methods for assessing tau pathology in AD. It remains to be determined whether a combination of these metrics will help explain the diversity of AD presentation.

### Supplementary Information

**Additional file 1:** Tables S1 and S2. See separate online file.

**Additional file 2: Figure S6 (a)**. Tau seeding map of NFT stage I. See separate online files. MR imaging of the brain was performed in a healthy volunteer. Segmentation of regions of interest (ROIs) within the brain was performed semi-automatically or by a board-certified neuroradiologist (F.F.Y.). The weighted average tau deposition from all subjects within each NFT stage group was then applied to each ROI. Brain regions were included only if at least one subject within each NFT stage had positive tau seeding results. The ROIs were then visualized within a 3D projection of the volunteer’s brain. For clarity, 3D representation is limited to one hemisphere. A graded color scale from white to yellow to orange to red (the same color coding as used in Fig. 3) indicates increasing amount of seeding. Brain regions not sampled in this study are colored in grey. Note that many brains regions that are traditionally not included in AD pathology staging exhibit tau seeding, some of them at early NFT stages.

**Additional file 3: Figure S6 (b)**. Tau seeding map of NFT stage III. See separate online files. MR imaging of the brain was performed in a healthy volunteer. Segmentation of regions of interest (ROIs) within the brain was performed semi-automatically or by a board-certified neuroradiologist (F.F.Y.). The weighted average tau deposition from all subjects within each NFT stage group was then applied to each ROI. Brain regions were included only if at least one subject within each NFT stage had positive tau seeding results. The ROIs were then visualized within a 3D projection of the volunteer’s brain. For clarity, 3D representation is limited to one hemisphere. A graded color scale from white to yellow to orange to red (the same color coding as used in Fig. 3) indicates increasing amount of seeding. Brain regions not sampled in this study are colored in grey. Note that many brains regions that are traditionally not included in AD pathology staging exhibit tau seeding, some of them at early NFT stages.

**Additional file 4: Figure S6 (c)**. Tau seeding map of NFT stage V. See separate online files. MR imaging of the brain was performed in a healthy volunteer. Segmentation of regions of interest (ROIs) within the brain was performed semi-automatically or by a board-certified neuroradiologist (F.F.Y.). The weighted average tau deposition from all subjects within each NFT stage group was then applied to each ROI. Brain regions were included only if at least one subject within each NFT stage had positive tau seeding results. The ROIs were then visualized within a 3D projection of the volunteer’s brain. For clarity, 3D representation is limited to one hemisphere. A graded color scale from white to yellow to orange to red (the same color coding as used in Fig. 3) indicates increasing amount of seeding. Brain regions not sampled in this study are colored in grey. Note that many brains regions that are traditionally not included in AD pathology staging exhibit tau seeding, some of them at early NFT stages.

## Data Availability

The datasets used and/or analyzed during the current study are available from the corresponding author by reasonable request.
