# Correction to: Anatomic survey of seeding in Alzheimer’s disease brains reveals unexpected patterns

**DOI:** 10.1186/s40478-024-01795-y

**Published:** 2024-06-04

**Authors:** Barbara E. Stopschinski, Kelly Del Tredici, Sandi‑Jo Estill‑Terpack, Estifanos Ghebremedhin, Fang F. Yu, Heiko Braak, Marc I. Diamond

**Affiliations:** 1 Center for Alzheimer’s and Neurodegenerative Diseases, Peter O’Donnell Jr. Brain Institute, NL10.120, University of Texas Southwestern Medical Center, Harry Hines Blvd., 6000, Dallas, TX 75390 USA; 2 Clinical Neuroanatomy Section/Department of Neurology, Center for Biomedical Research, University of Ulm, Ulm, Germany; 3 Institute of Clinical Neuroanatomy, J. W. Goethe University, Frankfurt am Main, Germany; 4 Department for Radiology, Neuroradiology Division, University of Texas Southwestern Medical Center, Dallas, TX USA

**Correction to**: ***acta neuropathol commun*9, 164 (2021).**

10.1186/s40478-021-01255-x

Following publication of the original article [1], the authors identified an error in the author name of Estifanos Ghebremedhin. The Family Name contained an error.

The incorrect author name is: Estifanos Ghebremdehin.

The correct author name is: Estifanos Ghebremedhin.

The author group has been updated above and the original article [1] has been corrected.
