# Supplementary material for: Anatomic survey of seeding in Alzheimer’s disease brains reveals unexpected patterns
Source: Acta Neuropathol Commun. 2021 Oct 11;9:164. doi: 10.1186/s40478-021-01255-x (PMC8507321; doi:10.1186/s40478-021-01255-x)
Supplement: Supplementary file 1 — Additional file 1: Tables S1 and S2. See separate online file. [file 40478_2021_1255_MOESM1_ESM.docx]

**Additional files**

**Table S1**

| **NFT I** | |  |  |  |  |  |  |  |  |  |
| --- | --- | --- | --- | --- | --- | --- | --- | --- | --- | --- |
|  |  | **Brain regions** | | | | | | | |  |
| **Individual** | **Seeding** | **1** | **12** | **15** | **18** | **19** | **20** | **21** | **22** |  |
| 1 | 0.01 | ++ |  |  |  |  |  |  |  | 9 positive neurons |
| 2 | 0.04 |  | axons  + |  |  |  |  |  |  | GP neurons negative |
| 2 | 0.22 |  |  | axons  ++ |  |  |  |  |  | n/a |
| 3 | 0.08 | + |  |  |  |  |  |  |  | 1 positive neuron |
| 3 | 0.07 |  |  |  | + |  |  |  |  | PG neurons positive |
| 4 | 0.14 |  |  |  |  | 0 |  |  |  | IO neurons, axons negative, IRZ + neurons, axons |
| 4 | 0.13 |  |  |  |  |  | + |  |  | 1 positive axon but in the emboliform nucleus rather than DN |
| 5 | 0.33 |  |  | axons  + |  |  |  |  |  | n/a |
| 6 | 0.19 |  |  | axons  + |  |  |  |  |  | 1 positive neuron |

| **NFT III** | |  |  |  |  |  |  |  |  |  |
| --- | --- | --- | --- | --- | --- | --- | --- | --- | --- | --- |
|  |  | **Brain regions** | | | | | | | |  |
| **Individual** | **Seeding** | **1** | **12** | **15** | **18** | **19** | **20** | **21** | **22** |  |
| 7 | 2.65 |  | axons  +++ |  |  |  |  |  |  | GP neurons negative |
| 7 | 0.79 |  |  |  | n/a |  |  |  |  | dorsal raphe +(+) punched in error instead of PG |
| 7 | 0.60 |  |  |  |  | axons  + |  |  |  | IO neurons negative, ARTAG perivascular, IRZ +++ neurons, axons |
| 9 | 0.15 |  |  |  | + |  |  |  |  | PG neurons positive |
| 9 | 1.74 |  |  |  |  |  | * |  |  | CC negative, Bergmann glia positive* |
| 9 | 0.25 |  |  |  |  |  |  | + |  | DN neurons and axons + |
| 10 | 1.38 |  | axons  ++ |  |  |  |  |  |  | GP neurons negative |
| 10 | 0.05 |  |  |  |  |  |  |  | axons  + | n/a |
| 12 | 0.44 |  | axons  + |  |  |  |  |  |  | GP neurons negative, ARTAG +++ |
| 12 | 5.47 |  |  | +++ |  |  |  |  |  | SN axons and neurons positive +++ |
| 12 | 0.08 |  |  |  | +(+) |  |  |  |  | PG neurons and 5 axons positive |
| 12 | 2.29 |  |  |  |  | axons  ++ |  |  |  | IO neurons negative, ARTAG not only perivascular +++,  IRZ neurons, axons +++ |
| 12 | 1.39 |  |  |  |  |  | * |  |  | CC negative, Bergman glia positive* |
| 12 | 0.60 |  |  |  |  |  |  | axons  ++ |  | DN neurons ++ |
| 12 | 8.48 |  |  |  |  |  |  |  | axons  + | IC axons mild + despite strong signal |
| 13 | 0.13 |  |  | axons  ++ |  |  |  |  |  | SN neurons negative |

| **NFT V** | |  |  |  |  |  |  |  |  |  |
| --- | --- | --- | --- | --- | --- | --- | --- | --- | --- | --- |
|  |  | **Brain regions** | | | | | | | |  |
| **Individual** | **Seeding** | **1** | **12** | **15** | **18** | **19** | **20** | **21** | **22** |  |
| 14 | 0.78 |  | axons  ++ |  |  |  |  |  |  | GP neurons negative |
| 14 | 0.11 |  |  |  | 0 |  |  |  |  | n/a |
| 14 | 0.96 |  |  |  |  | axons  + |  |  |  | IO neurons negative, IRZ neurons/axons +++ |
| 14 | 0.04 |  |  |  |  |  |  | axons  ++ |  | DN neurons negative, DN axons ++ despite weak signal |
| 14 | 0.14 |  |  |  |  |  |  |  | axons  ++ | IC axons ++ despite weak signal |
| 15 | 0.08 |  |  |  |  | 0 |  |  |  | IO negative, IRZ + |
| 16 | 1.25 |  | axons  + |  |  |  |  |  |  | GP neurons negative |
| 16 | 1.07 |  |  |  | axons  ++ |  |  |  |  | PG neurons positive ++ |
| 16 | 0.24 |  |  |  |  | axon  + |  |  |  | IO neurons negative, IO 1 positive axon/ARTAG perivascular +, IRZ neurons/ axons ++ |
| 16 | 0.05 |  |  |  |  |  | 0 |  |  | CC |
| 16 | 2.24 |  |  |  |  |  |  | axons  ++ |  | DN 6 neurons ++ |
| 17 | 4.32 |  | axons  ++ |  |  |  |  |  |  | GP neurons negative |
| 17 | 0.04 |  |  |  | 0 |  |  |  |  | n/a |
| 17 | 4.74 |  |  |  |  | axons  ++ |  |  |  | IO neurons negative, IRZ neurons/ axons +++ |
| 17 | 0.05 |  |  |  |  |  |  | 0 |  | n/a |
| 17 | 3.73 |  |  |  |  |  |  |  | axon  + | 1 positive axon,  despite signal strength |
| 19 | 6.88 |  | axons  ++ |  |  |  |  |  |  | GP neurons negative |
| 19 | 0.24 |  |  |  | 0 |  |  |  |  | PG neurons and axons negative |
| 19 | 0.28 |  |  |  |  | 0 |  |  |  | IO neurons negative, IO axons negative, IRZ axons + |
| 19 | 0.35 |  |  |  |  |  |  |  | axons  ++ | n/a |
| 20 | 0.78 |  | axons  ++ |  |  |  |  |  |  | GP neurons negative |
| 20 | 0.64 |  |  |  | axon  + |  |  |  |  | PG neurons negative, 1 positive axon |
| 20 | 0.25 |  |  |  |  | axons  + |  |  |  | IO neurons negative, IO axons +, IRZ neurons/axons +++ |

**Table S1. Seeding and neuropathology in selected cases. 1** – transentorhinal cortex (TRE); **12** – globus pallidus (GP); **15** – substantia nigra, pars compacta (SNpc); **18** – pontine gray (PG); **19** – inferior olivary nucleus (IO); **20** – cerebellar cortex (CC); **21** – cerebellar dentate nucleus (DE); **22** – internal capsule (IC). 0 – no AT8-positivity; + – a few (1-4) AT8-positive neurons or axons; ++ – AT8-positive neurons and/or axons (moderate); +++ – AT8-positive neurons and/or axons (numerous); * – ARTAG, e.g., perivascular tau-positive astrocytes, thorn-shaped astrocytes; IRZ – medullary intermediate reticular zone; n/a – not applicable.

**Table S2**

| **Brain region** | **Expectation** | **Reference for expectation** | **Findings** | **Comments** |
| --- | --- | --- | --- | --- |
| 1 - TRE | Seeding in all cases | Kaufmann et al., 2018 | Unexpected => n=2/6 cases at NFT stage I did not show seeding | Both seeding-negative cases displayed AT8-positive neurons |
| 2 - EC | Seeding starting at early NFT stages (I-II) | Kaufmann et al., 2018 | As expected, seeding in n=2/6 NFT stage I cases and all NFT stage III and V cases |  |
| 3 - CA1 | Seeding starting at early NFT stages (I-II) | Kaufmann et al., 2018 | As expected, seeding in n=2/6 NFT stage I cases and all NFT stage III and V cases |  |
| 4 - AMY | Seeding starting at late NFT stages | Braak et al., 1991 | Unexpected => seeding in n=1/6 NFT stage I case, in n=6/7 NFT stage III cases and all NFT stage V cases |  |
| 5 - STG | Seeding starting at early/ intermediate NFT stages (II-III) | Kaufmann et al., 2018 | As expected, seeding in n=3/7 NFT stage III cases and in all NFT stage V cases |  |
| 6 - TTG | Seeding starting at late NFT stages (V-VI) | Braak et al, 1991 | Unexpected => seeding in n=1/7 NFT stage III case; expectedly in n=6/7 NFT stage V cases |  |
| 7 - PV | Seeding starting at intermediate/ late NFT stages (III-VI) | Kaufmann et al., 2018 | As expected, seeding was seen in only n=1/7 NFT stage III case and some NFT stage V cases (n=4/7) |  |
| 8 - PS | Seeding starting at intermediate NFT stages (III-IV) | Braak et al, 1991 | As expected, seeding in n=3/7 NFT stage III cases and in all NFT stage V cases |  |
| 9 - ACC | Seeding starting at intermediate NFT stages (III-IV) | Braak et al, 1991 | As expected, seeding in n=5/7 NFT stage III cases and in all NFT stage V cases |  |
| 10 - RSC/PSC | Seeding starting at intermediate/ late NFT stages (III-VI) | Braak et al, 1991 | As expected, seeding in n=2/7 NFT stage III cases and in all NFT stage V cases |  |
| 11 - PUT | Seeding starting at intermediate/ late NFT stages (III-VI) | Braak et al, 1991 | As expected, seeding in n=1/7 NFT stage III case and in n=6/7 NFT stage V cases |  |
| 12 - GP | Seeding not expected since it typically does not develop tau pathology (in contrast to Aβ plaques) in AD | Braak and Del Tredici, 2015 | Unexpected => seeding in n=3/7 NFT stage III cases and in n=6/7 NFT stage V cases | AT8 IHC in 9 cases showed AT8-positive axons plus ARTAG.  Note: The AT8-positive axons in the GP likely originate from the Meynert’s basal nucleus (BN) since BN seeding levels in all of but one case (case 10) are higher than in their respective GP punches. |
| 13 - MD | Seeding starting at intermediate/ late NFT stages (III-V) | Braak et al, 1991 | As expected, seeding in n=2/7 NFT stage III cases and in all NFT stage V cases |  |
| 14 - OFC | Seeding starting at intermediate NFT stages (III-IV) | Braak et al, 1991 | As expected, seeding in n=4/7 NFT stage III cases and in all NFT stage V cases |  |
| 15 - SN | Seeding starting at late NFT stages (V-VI) | Braak et al, 1991 | Unexpected => seeding seen earlier – in most NFT stage III cases (n=6/7) and in all NFT stage V cases | AT8 IHC in 5 cases showed AT8-positive axons. Possible region of origin could be LC. |
| 16 - LC | Seeding starting at early NFT stages (I and II | Kaufmann et al., 2018 | As expected, seeding in n=1/6 NFT stage I case and in all NFT stage III and V cases |  |
| 17 - BN | Seeding starting at intermediate NFT stages (III-IV) | Braak et al, 1991 | As expected, seeding in n=4/7 NFT stage III cases and in all NFT stage V cases | Seeding was always higher than respective GP case (with one exception: case 10), suggesting that AT8-positive axons in GP originate from the BN |
| 18 - PG | Seeding not expected | Braak et al, 1991 | Unexpected => seeding in n=1/7 NFT stage III case and in n=2/7 NFT stage V cases  Note: The dorsal raphe nucleus (DRN) in case 7 was punched accidentally and likely explains the seeding in this sample | AT8 IHC in 8 cases showed AT8-positive axons, neurons, and in some cases neurites (although AT8 positivity does NOT necessarily correlate with presence of seeding) |
| 19 - IO | Seeding not expected | Braak et al, 1991 | Unexpected => seeding in n=2/7 NFT stage III cases and in n=2/7 NFT stage V cases  Note: Some punches from IO were contaminated by overlapping AT8-positive neurons/axons in the IRZ | AT8 IHC of 9 cases showed AT8-positive axons and in some cases ARTAG |
| 20 - CC | Seeding not expected | Braak et al, 1991 | Unexpected => seeding in n=2/7 NFT stage III cases | AT8 IHC showed AT8-positive Bergmann glia, a new ARTAG finding |
| 21 - DN | Seeding not expected | Braak et al, 1991 | Unexpected => seeding in n=1/7 NFT stage III case and in n=1/7 NFT stage V case | AT8 IHC in 4 cases showed AT8-positive neurons and axons |
| 22 - IC | Seeding not expected | Braak et al, 1991 | Unexpected => seeding in n=1/7 NFT stage III case and in n=4/7 NFT stage V cases | AT8 IHC in 5 cases showed AT8-positive axons (although severity of AT8 pathology did not correlate with seeding signal) |
| 23 - TS | Seeding starting at intermediate NFT stages (III-IV) | Braak et al, 1991 | As anticipated, seeding in n=3/7 NFT stage III cases and in n=6/7 NFT stage V cases |  |
| 24 - OB | Seeding starting at an early/intermediate NFT stages (II-III) | Attems et al, 2005 | As expected, seeding in n=4/7 NFT stage III cases; however, unexpected => in only n=5/6 NFT stage V cases |  |
| 25 - OC | Seeding expected | DeVos et al, 2018 | Unexpected => seeding in only one individual (case 20) | Samples were not available from all cases for AT8 IHC. |

**Table S2. Expected and unexpected seeding results across brain regions.** Summary table of all seeding results based on expectations from seeding and/or neuropathology data from prior studies. For unexpected results, AT8 IHC was performed as listed in Table S1. Unanticipated seeding results are highlighted above in red.
